# Communicating the uncertainty in estimated greenhouse gas emissions from agriculture

**DOI:** 10.1016/j.jenvman.2015.05.034

**Published:** 2015-09-01

**Authors:** Alice E. Milne, Margaret J. Glendining, R. Murray Lark, Sarah A.M. Perryman, Taylor Gordon, Andrew P. Whitmore

**Affiliations:** a Rothamsted Research, Harpenden, Hertfordshire, AL5 2JQ, UK; b British Geological Survey, Nicker Hill, Keyworth, Nottingham, NG12 5GG, UK

**Keywords:** Uncertainty, Greenhouse gas emissions, Communication methods, Communicating statistics

## Abstract

In an effort to mitigate anthropogenic effects on the global climate system, industrialised countries are required to quantify and report, for various economic sectors, the annual emissions of greenhouse gases from their several sources and the absorption of the same in different sinks. These estimates are uncertain, and this uncertainty must be communicated effectively, if government bodies, research scientists or members of the public are to draw sound conclusions. Our interest is in communicating the uncertainty in estimates of greenhouse gas emissions from agriculture to those who might directly use the results from the inventory. We tested six methods of communication. These were: a verbal scale using the IPCC calibrated phrases such as ‘likely’ and ‘very unlikely’; probabilities that emissions are within a defined range of values; confidence intervals for the expected value; histograms; box plots; and shaded arrays that depict the probability density of the uncertain quantity. In a formal trial we used these methods to communicate uncertainty about four specific inferences about greenhouse gas emissions in the UK. Sixty four individuals who use results from the greenhouse gas inventory professionally participated in the trial, and we tested how effectively the uncertainty about these inferences was communicated by means of a questionnaire. Our results showed differences in the efficacy of the methods of communication, and interactions with the nature of the target audience. We found that, although the verbal scale was thought to be a good method of communication it did not convey enough information and was open to misinterpretation. Shaded arrays were similarly criticised for being open to misinterpretation, but proved to give the best impression of uncertainty when participants were asked to interpret results from the greenhouse gas inventory. Box plots were most favoured by our participants largely because they were particularly favoured by those who worked in research or had a stronger mathematical background. We propose a combination of methods should be used to convey uncertainty in emissions and that this combination should be tailored to the professional group.

## Highlights

• We tested 6 different methods for communicating uncertainty in greenhouse gases.
• Choice of method of communication should be influenced by professional background.
• The IPCC verbal scale has value but should be used alongside more numerate methods.
• Boxplots communicate uncertainty effectively to those familiar with this method.
• Shaded arrays communicate uncertainty effectively to most individuals.

## 1 Introduction

In an effort to mitigate anthropogenic effects on the global climate system, industrialised countries (known as Annex I countries) are required to make an inventory of annual emissions of greenhouse gases, and absorption of the same in various sinks, in different economic sectors and report this to the United Nations Framework Convention on Climate Change (UNFCCC). This inventory allows governments to track changes in emissions and so ensure that reductions are on track with agreed targets, and also allows research scientists, industry and members of the public to see how much the various sectors contribute to total emissions and so decide where mitigation effort should be best spent.

The estimated emissions are calculated using the models and guidance published by the Intergovernmental Panel on Climate Change (IPCC) (Eggleston et al., 2006). At their simplest, these models combine country specific activity data (for example cattle numbers) with IPCC emission factors. Estimates of emissions are uncertain both because of errors in the conceptualization of the model framework and because the model inputs (e.g. the activity data and emissions factors) are themselves uncertain. All Annex I countries are obliged, as far as possible, to quantify the uncertainties in their estimates of emissions by determining how uncertainties in the model inputs propagate through the model (Eggleston et al., 2006; Monni et al., 2007; Milne et al., 2014). To do this we treat the model inputs as random variables with distributions which are either derived from available data or are elicited from experts. Using Monte Carlo simulation, we sample from the distributions of all of the model inputs, then calculate emissions using the sampled values and so derive model outputs (see Milne et al., 2014). The model output is therefore a random quantity with a distribution. We regard this distribution of outputs as the basic representation of our uncertainty about the emissions that the model predicts.

It is important to report the uncertainty in the estimates of emissions because this information enables the users of the inventory to assess the reliability of estimates and allows them to determine whether significant reductions in emissions have been made. Without this understanding it is not possible to draw proper conclusions and so make sound decisions. Therefore the uncertainty in the estimated emissions must be effectively communicated. Communicating uncertainty is challenging. The everyday use of ‘uncertainty’ has negative connotations. The admission that scientific knowledge is uncertain may be interpreted popularly as: ‘scientists don't know what they are talking about’ (Talking Climate, 2013). This is a problem if important, though uncertain, information is consequently ignored in public debate and policy-making. Much research has been done on how to communicate the uncertainty in weather predictions, and medical information, and this has been reasonably successful (Zikmund-Fisher et al., 2008; Spiegelhalter et al., 2011). The methods used to communicate uncertainty generally depend on the subject matter and the background of the target audience.

Our interest is in communicating the uncertainty in estimates of greenhouse gas emissions from agriculture to those who directly use the results from the inventory. This includes UK government representatives (in England the Department for Environment, Food and Rural Affairs and analogue departments of the devolved administrations in Scotland, Wales and Northern Ireland), economists, representatives from non-government organisations with an environmental focus, research organisations, agricultural levy boards, and industry representatives. These individuals may in turn be required to communicate the uncertainty to other groups, such as farmers or the general public, but communicating to these groups was not our central concern.

Given our basic quantification of uncertainty, the output distribution, we can communicate the uncertainty in a number of ways. For example, we can present the distribution as a histogram, or an empirical probability density function (PDF). These methods are graphical. Graphics are used widely to communicate uncertainty and there are various types. For example, the graphic that we call a ‘shaded array’ in this study portrays the PDF by a shaded bar. The density of shading at a position on the bar is proportional to the probability density at that value of the variable. This graphic was used effectively in the DESSAC decision support system for arable crops to present the uncertainty in yield estimates (Parsons et al., 2009), and is similar to the fan chart used by the Bank of England to show predicted economic growth (Spiegelhalter et al., 2011). Spiegelhalter et al. (2011) reviewed how graphics can be used to convey uncertainty to a general audience. They explain that the most suitable choice of visualization depends on the objectives of the presenter, the context of the communication, and the audience.

Alternatively, or in addition to graphical methods, we may characterize the uncertainty numerically, for example as a probability interval for the model output's distribution. This interval is defined by two percentiles of the empirical distribution, *P*_*L*_ and *P*_*U*_. Given the uncertainty in those aspects of the model which we treat as uncertain, and conditional on the assumption that other aspects of the model are sound, the probability interval is therefore an interval within which we expect to find the quantity predicted by the model with a probability of *P*_*U*_−*P*_*L*_%. This probability interval is called a 95% confidence interval in the IPCC manual, in the case where *P*_*U*_ is set to 97.5 and *P*_*L*_ to is set to 2.5 (Eggleston et al., 2006) and we follow that convention.

Uncertainty can also be simply described using words, for example, on a verbal scale. Words can be adapted to any level of understanding, and for most, the message they convey can be easily remembered. Words are often used to convey the uncertainty of events, for example, weather forecasters might tell us that snow is *likely*, and health workers might tell us that smoking is *very likely* to damage our health. Words can be straightforward to understand, but the transfer of information from a numerical to a verbal scale inevitably loses information, and so the result may lack precision. This method of communicating uncertainty is primarily criticized because of its ambiguity and the fact that verbal information may be interpreted inconsistently by different individuals (Kloprogge et al., 2007; Spiegelhalter et al., 2011). In an attempt to overcome this, the IPCC (Mastrandrea et al., 2010) introduced a verbal scale in which particular ranges of probabilities correspond to ‘calibrated phrases’, for example an event which is expected to occur with a probability of more than 99% is said to be ‘virtually certain’. This scale is enhanced by some authors with the use of a traffic light scheme colour code, whereby the most uncertain phrases are linked to red, and the least uncertain green (Table 1). The verbal scale has been criticized, as studies have shown that it is not always interpreted consistently (Budescu et al., 2009; Harris and Corner, 2011).

To establish how best to communicate the uncertainty in emissions estimates to the users of the inventory, we tested six methods of communication. These were words (a verbal scale in the form of calibrated phrases), probabilities, confidence intervals, histograms, box plots and shaded arrays. We used all these methods to present uncertainty about information concerning four particular questions about greenhouse gas emissions in the UK to 64 individuals who use results from the greenhouse gas inventory professionally. We then recorded their opinions about how effectively these methods communicated uncertainty. We present the results of this study and show that responses are influenced by both professional background and the level to which individuals were educated in mathematics. Based on our results we propose some guidelines for reporting uncertainty to various groups who might use the results of the greenhouse gas inventory.

## 2 Material and methods

We held three workshops at which the participants were invited to answer questions on six different methods of communicating the uncertainty in greenhouse gas emissions. In total 64 individuals took part. Each workshop followed the same format and was attended by participants from various professional backgrounds.

Each workshop began with an introductory talk in which we explained why estimates of greenhouse gas emissions were uncertain and that it was important to communicate this effectively. We explained that we would present estimates of emissions using six different methods and that we wanted the participants to complete a questionnaire on how effective they thought each method was. We then showed the participants the questionnaire format and explained how it should be filled in. We did not disclose any of the methods at this point. We emphasised that whilst we were happy for the participants to talk to each other during the process, it was important that they gave us their opinion and not the opinion that they felt was most commonly shared.

After the introductory talk we gave each participant a copy of the questionnaire and directed them to a room where the six methods of communication were displayed on six posters (one for each method). The participants were told the order that they should visit each poster. This order was randomised to avoid any bias caused by the participants finding a particular method easier to interpret because they had seen the same material presented in a different format previously. This phenomenon is exploited in progressive disclosure, a technique whereby individuals are gradually presented with information of increasing difficulty and so are not overwhelmed by difficult concepts from the start. The participants were given an hour and a half to complete the questionnaire. Stewards were positioned at each poster to help the participants with any problems that they had with understanding the questionnaire, but they were not permitted to explain the methods of communication.

### 2.1 The test material

The methods of communicating uncertainty that we chose to test were a verbal scale, probabilities, confidence intervals, histograms, box plots and shaded arrays. We could have chosen to fit PDFs to the Monte Carlo simulation outputs, and used those to communicate uncertainty. Probability density functions are, in appearance, very similar to histograms, and we did not have the resources to test more than six methods. Some initial testing showed that end-users found the histogram, which displays the frequency of observations, more intuitive than the PDF which shows probability density. Therefore we chose to use histograms over PDFs. To test our six methods of communication we considered four scenarios in which an inventory user might use the inventory results. These were:

Scenario A: To compare emissions from various sectors or countries
Scenario B: To compare emissions to a given reference value
Scenario C: To assess whether emissions have diminished
Scenario D: To assess the effectiveness of a given mitigation method

**Fig. 1:**
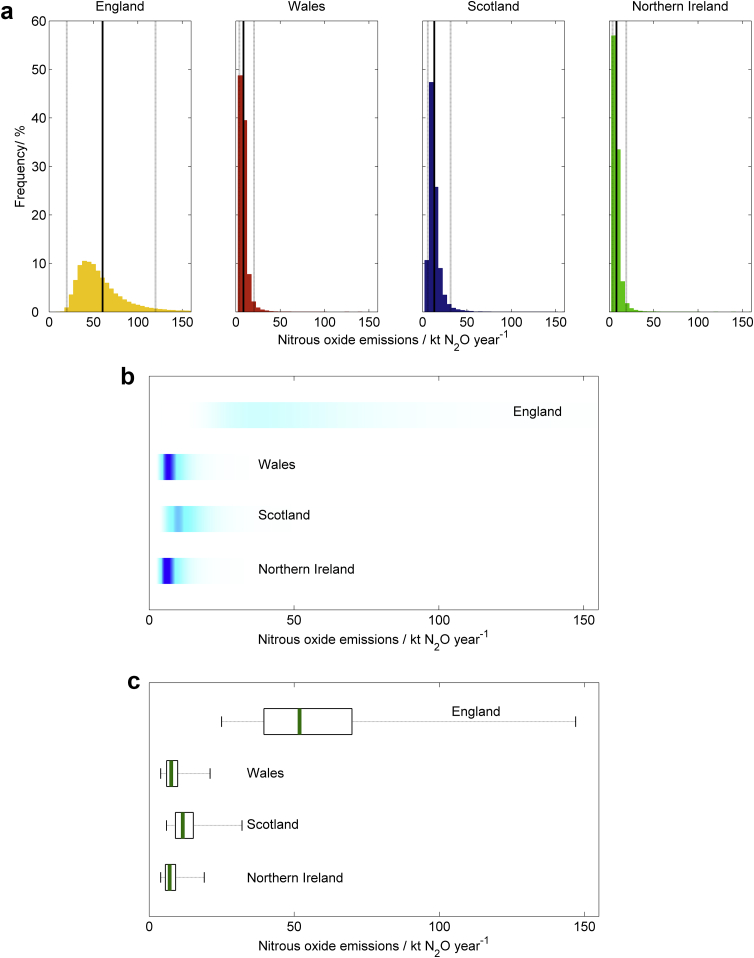
Scenario A: Comparing emissions of nitrous oxide from different countries. The estimated emissions of N_2_0 from agriculture in 2010 presented with (a) histograms, with the means shown by the solid black lines and the 95% confidence interval shown by the solid grey lines, (b) shaded arrays, where the intensity of colour indicates the density of the underlying PDF, and (c) boxplots, where the green lines show the median values, the black boxes depicting the lower and upper quartiles and the dotted lines show the extent of the 95% confidence intervals. (For interpretation of the references to colour in this figure legend, the reader is referred to the web version of this article.).

**Fig. 2:**
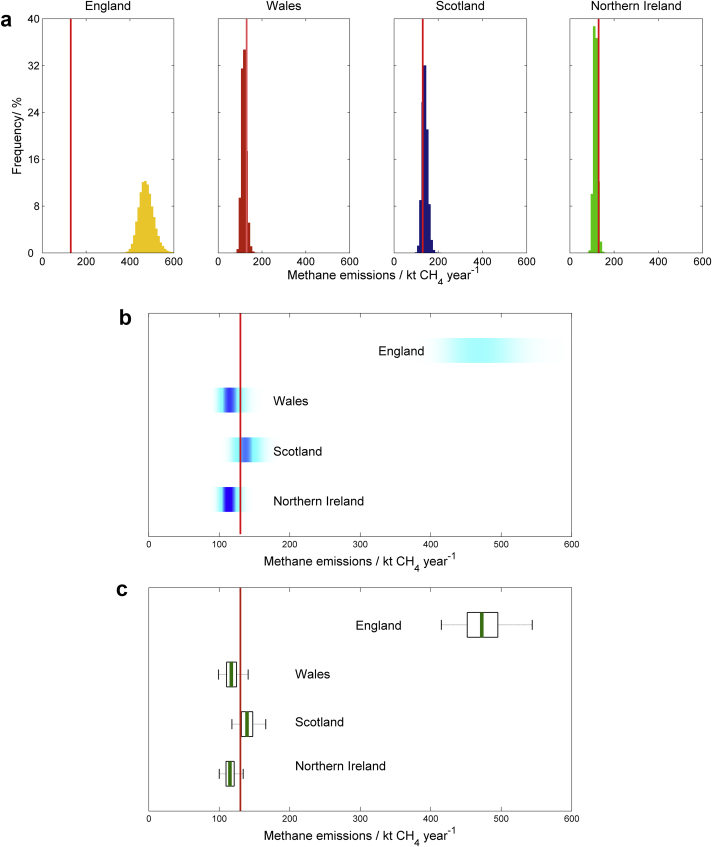
Scenario B: Comparing emissions of methane to a given threshold value. Estimated emissions of CH_4_ from agriculture in 2010 compared with an arbitrarily chosen threshold value of 130 kt CH_4_ year^−1^, presented with (a) histograms, (b) shaded arrays, where the intensity of colour indicates the density of the underlying PDF, and (c) boxplots, where the green lines show the median values, the black boxes depicting the lower and upper quartiles and the solid grey lines show the extent of the 95% confidence intervals. In each case the threshold value is marked by the solid red line. (For interpretation of the references to colour in this figure legend, the reader is referred to the web version of this article.).

**Fig. 3:**
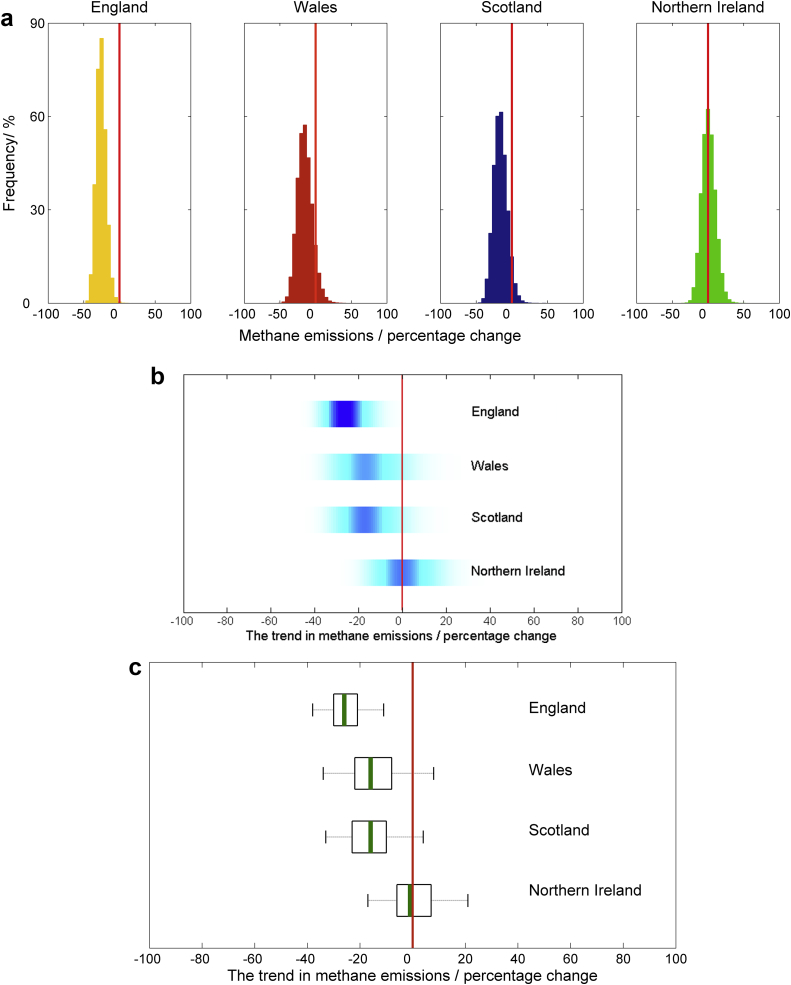
Scenario C: Assessing changes in methane emissions over time. The trend in emissions of CH_4_ from agriculture between 1990 and 2010, presented with (a) histograms, (b) shaded arrays, where the intensity of colour indicates the density of the underlying PDF, and (c) boxplots, where the green lines show the median values, the black boxes depicting the lower and upper quartiles and the dotted lines show the extent of the 95% confidence intervals. In each case the zero line is marked by the solid red line. (For interpretation of the references to colour in this figure legend, the reader is referred to the web version of this article.).

**Fig. 4:**
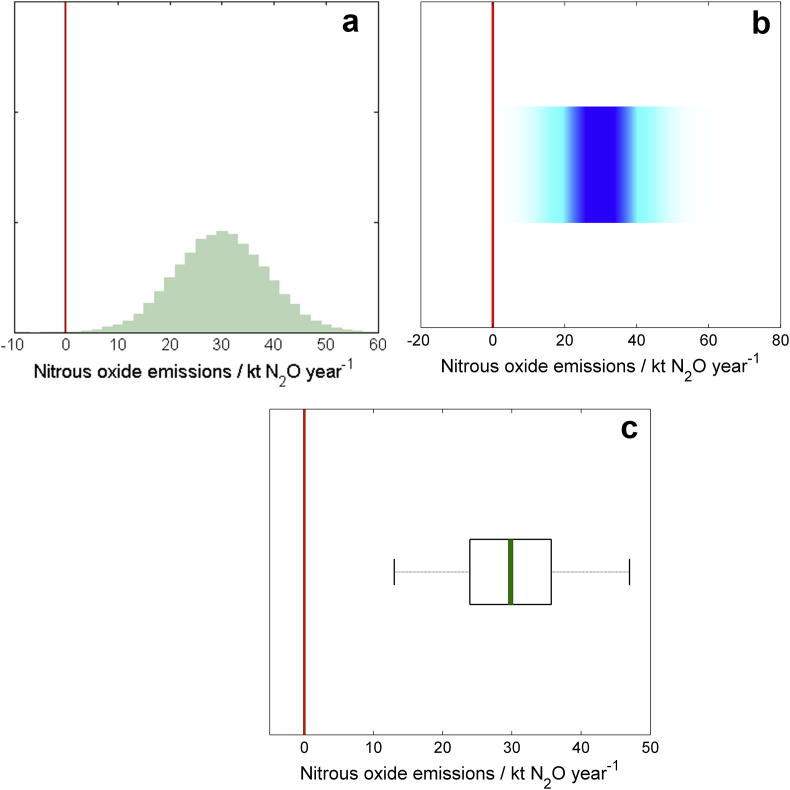
Scenario D: Assessing mitigation methods for the best opportunity to reduce emissions of nitrous oxide. The reduction in emissions of N_2_0 from English grasslands resulting from a mitigation strategy, presented with (a) histograms, (b) shaded arrays, where the intensity of colour indicates the density of the underlying PDF, and (c) boxplots, where the green lines show the median values, the black boxes depicting the lower and upper quartiles and the dotted lines show the extent of the 95% confidence intervals. The solid red line indicates no reduction in emissions. (For interpretation of the references to colour in this figure legend, the reader is referred to the web version of this article.).

We presented information from the Monte Carlo simulation samples of model output for each of the four scenarios listed above using our six methods of communication (Boxes 1–3 and Figs. 1–4). All methods of communication are based on the distribution of relevant values drawn from the Monte Carlo simulation sample of model outputs. The same set of four scenarios was used to test each method. For the first scenario we presented estimates of nitrous oxide emissions from agriculture for each of the four countries in the UK (England, Wales, Scotland and Northern Ireland). For the second, we showed results on methane emissions from each country in the UK and compared these to an arbitrarily chosen reference value. For the third we showed the estimated trend in methane emissions from each country between the years 1990 and 2010. For the fourth we presented the estimated nitrous oxide emissions from grasslands both with and without a mitigation strategy applied.

#### 2.1.1 Verbal scale

We used the verbal scale proposed by the IPCC (Table 1) to communicate the uncertainty in the results presented for scenarios B, C and D. In support of this method we colour coded the calibrated phrases (Kloprogge et al., 2007). The IPCC calibration is not suitable for describing the uncertainty in a given estimate of emissions, as for example we present for scenario A. The 95% confidence interval for these estimated emissions were all large (approximately −60% and +100% of the mean) and so for this scenario we simply stated that the emissions were all *very uncertain.*

#### 2.1.2 Probabilities

Probabilities can be estimated from the Monte Carlo simulation outputs that describe the uncertainty about the statements the participants are asked to consider. For example, if 95% of the Monte Carlo simulations showed that methane emissions from agriculture in England were smaller than the reference value then the probability that this is the case is estimated as 95%.

#### 2.1.3 Confidence intervals

The IPCC manual calls the probability interval defined by the 2.5 and 97.5 percentile the 95% confidence interval. The percentiles were computed from the Monte Carlo simulation output. For example, 2.5% of the Monte Carlo simulations were smaller than the 2.5 percentile. We presented the confidence interval both in the same units as the expected value and as a percentage of the expected value.

#### 2.1.4 Histograms

The histogram graphically represents the distribution of the Monte Carlo simulation output. The outputs are divided into several classes, known as bins, all with equal width. The histogram is a graph of the number of outputs in each class (the frequency). This is different from a PDF because the area of the histogram equals the number of observations.

#### 2.1.5 Shaded arrays

The shaded array portrays the PDF of the Monte Carlo simulation output by a shaded bar. The density of shading at a given value on the bar corresponds to the density of the PDF at a particular value.

#### 2.1.6 Boxplots

Boxplots offer another way of graphically showing the PDF of the Monte Carlo simulation. The box encloses the interquartile range, the median is marked by a line within the box and the ‘whiskers’ extend from the interquartile range to the 2.5 and 97.5 percentiles.

### 2.2 Questionnaire

The participants assigned themselves to one of four ‘professional’ groups depending on occupation. These were (1) ‘Government and policy’ which included government representatives; (2) ‘Industry’ which included representatives from levy boards, the UK National Farmers Union, and agricultural manufacturers; (3) Research organisations, and (4) ‘Environment’ which included organisations such as those responsible for calculating farm carbon footprints. They were then asked to record their level of mathematical education as (i) ‘lower’ which equates to compulsory education in mathematics to the age of approximately 16; (ii) ‘higher’ which equates to education in mathematics to the age of approximately 18; or (iii) ‘degree level’ which equates to degree level and above.

The questionnaire had four central questions. The questions were asked in ‘closed form’ (i.e. the participants were asked to tick the response that most closely represented their thoughts) so that we could analyse the results statistically. There was also room for the participants to write additional comments. Questions 1 and 2 were asked for each of the six methods. Questions 3 and 4 were asked for all methods except for *the verbal scale* as there was insufficient information given to answer these questions using that method.

*Question 1: Is the information presented on uncertainty sufficient for your needs?* Answer selected from the following three responses: 1) Not enough information; 2) Shows the information I want; or 3) More information than I want or need.
*Question 2: Is this method of representing uncertainty straightforward to interpret?* Answer selected from the following five responses: 1) I find it impossible to understand; 2) I understand most of what has been presented but it took me a while to get it; 3) I think this method could be misinterpreted (please expand below); 4) Good but needs more explanation (please expand below) or 5) The message is clear.
*Question 3:* Is the following statement about Scenario A clear from the poster “The estimated emissions are most uncertain for England” Answer Yes or No.
*Question 4:* Is the following statement about Scenario C clear from the poster? “It is more uncertain that emissions from Scotland have reduced than that emissions from England have reduced” Answer Yes or No.

Our questionnaire aimed to evaluate how well each method communicated uncertainty to the various groups. We did not want the participants to feel that it was a test of their ability. Therefore we did not ask the participants to directly interpret the results on the posters, but in Questions 3 and 4 we did ask if certain statements about the results were clear. See S1 for the full layout of Q1–4.

We asked the participants to identify which method or methods they found best for communicating uncertainty (Question 5) and which method or methods they would choose to communicate uncertainty to other groups that they work with (Question 6). There were additional questions about specific methods. We asked if colour coding with words aided interpretation. We also tested to see if the perceptions of the IPCC phrases mapped to the probabilities they represented by asking the participants to write down the probability range between 0 and 100% that they thought mapped to each phrase. We asked whether it was helpful to have confidence intervals expressed in the same units as the mean or as a percentage of the mean. Finally we asked the participants to vote for the method that they thought communicated uncertainty the best.

### 2.3 Method of analysis

We analysed the responses to questions 1–4 in two ways. In the first analyses we considered differences between the methods of communication over the different scenarios. We present the results in contingency tables in which the rows are responses to the questions and the columns are the scenarios (A–D) within communication method. The contingency table for Question 1 is shown in Table 2a. Under our null hypothesis the responses are independent of the scenario and method, and so the same distribution of responses is expected for each method–scenario combination. Under the null hypothesis the expected number of responses in a cell is the product of the respective marginal (row and column) totals divided by the total number of responses in the table. If the expected number of responses in the *i* th cell (out of N) is *e*_*i*_ and the observed number is *o*_*i*_ we then compute a statistic to measure the evidence against the null hypothesis. In principle under the null hypothesis, and with *n*_*r*_ rows and *n*_*c*_ columns in the table, *X*^2^ is distributed by *χ*^2^ with (*n*_*c*_−1)( *n*_*r*_−1) degrees of freedom, but the fact that *o*_*i*_ is an integer introduces an approximation when the *o*_*i*_ over many cells is small. For this reason we obtain a *p* value for the *X*^2^ under the null hypothesis by the permutation method (Payne, 2011). We then considered a table in which the responses for each method are pooled, we call this the table pooled by method (Table 2b illustrates this for Question 1). The null hypothesis for this table is that over all methods the distributions of response to the question do not differ between scenarios. This is tested by computing *X*^2^ in the same way as above. Finally, we form sub-tables of the full table for each scenario (Table 2c illustrates this for Scenario A). Here the null hypothesis is that, for the scenario, the distributions of response are the same for each method.

$$X^{2}=\sum_{i=1}^{N}{\left(o_{i}-e_{i}\right)}^{2}/e_{i}$$

In the second set of analyses we consider the differences between either the professional groups or the level of mathematical education, for the different scenarios but considering each method of communication separately. Table 3a shows the full table for responses to Question 1 about the verbal scale. In this example we consider the scenarios with mathematical group, then pool by mathematical group (Table 3b) to compare the scenarios for Question 1 on the verbal scale. For each scenario there is then a sub-table in which the columns of the contingency table are mathematical groups (Table 3c).

## 3 Results

**Fig. 5:**
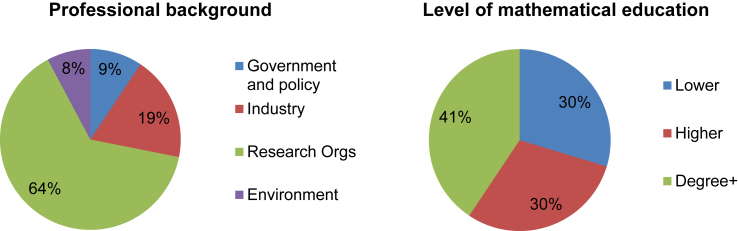
The percentage of participants from each professional and mathematical group.

Sixty four percent of our participants were from Research organisations and less than 10 percent were from each of Government and Environment. There was a reasonably even spread of mathematical education with approximately a third of participants in each group (Fig. 5).

### 3.1 Question 1: is the information presented on uncertainty sufficient for your needs?

**Fig. 6:**
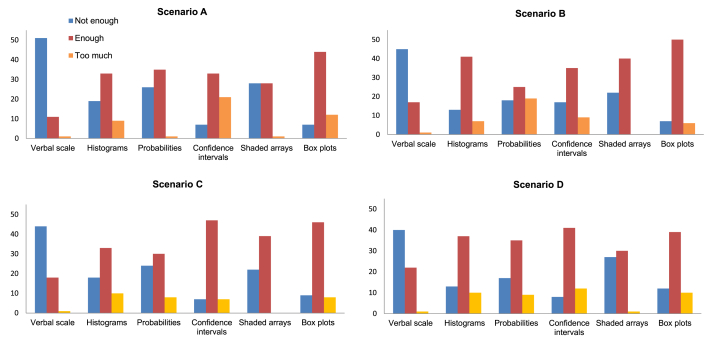
Bar charts showing how participants responded to Question 1 for each of the four scenarios.

Fig. 6 summarises the responses to Question 1. There were significant differences in how the participants responded to each method and scenario combination (Table 4 – Full table). When responses were pooled over the methods there were no significant differences however, showing that the overall effect is due to between-method differences. For each scenario sub-table the null hypothesis can be rejected. The deviation from expectation under the null hypothesis showed that more respondents than expected thought the verbal scale did not convey sufficient information, but that box plots conveyed sufficient.

The analyses of the responses according to professional group and scenario, for each method separately, showed few significant differences (Table S2). Only for confidence intervals was the null hypothesis for the full table rejected. There were significant differences between the responses to confidence intervals for the different scenarios when professional groups were pooled, in particular under scenario B more respondents than expected said that confidence intervals did not provide sufficient information. Analysis of the sub-tables for confidence intervals showed that under scenario C more members of the Government Policy group than expected under the null hypothesis thought confidence intervals did not give enough information.

For probabilities the null hypothesis was rejected for the table pooled by groups: more participants than expected stated that there was not enough information for scenario A, and too much for scenario B. The analysis of sub-tables for each scenario showed no differences between the professional groups for each scenario. The analysis of the scenario sub-tables showed a significant difference in how participants responded to boxplots under scenario B: the participants from industry stated that boxplots did not given enough information.

The analysis of the responses according to mathematical group (Table S3) showed that more participants than expected from the *lower* group found the information portrayed in the verbal scale sufficient for scenario A. For shaded arrays, the analysis of sub-tables showed a larger number of participants than expected with *higher* level mathematics stated that shaded arrays did not give enough information for scenarios A and D.

### 3.2 Question 2: is this method of representing uncertainty straightforward to interpret?

**Fig. 7:**
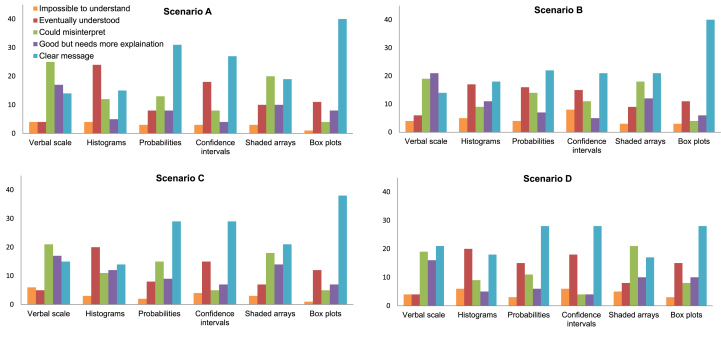
Bar charts showing how participants responded to Question 2 for each of the four scenarios.

The χ^2^-permutation test shows that there were significant differences in how the participants responded to each method when grouped by methods within scenarios, there were no significant effects of scenarios when methods were pooled but significant differences between methods within each scenario separately (Table 5). For all of the scenario sub-tables (Fig. 7), more respondents than expected stated that the verbal scale was open to misinterpretation or in need of more explanation. Shaded arrays were criticised by many as being open to misinterpretation. More respondents than expected found histograms most challenging to interpret, stating that they ‘eventually understood’. For probabilities, confidence intervals and boxplots the largest proportions of respondents stated that they gave a clear message.

When we analysed the data according to professional group and scenario (see Table S4) we found that the overall preference for boxplots was driven by the research scientists (our largest group, 85% of whom were classified as *higher* or *degree* in maths). The majority of research scientists found the message given by box plots was ‘clear’. For other groups there was no consistent view on boxplots. This pattern was shown to be significant across scenarios A–C (Table S4). Similarly, a greater number of research scientists than expected under the null hypothesis stated confidence intervals gave a clear message under scenario C.

When we analysed the data according to mathematical group and scenario (Table S5) we found the opinions on boxplots divided. The majority of respondents with *higher* or *degree* level maths found boxplots clear to interpret, whereas the responses of those with *lower* maths were mostly split between ‘eventually understood’ and ‘clear’. These differences were significant for scenarios A–C (Table S5). There was also a significant difference in response to confidence intervals between mathematical groups under scenario C. The majority of respondents with *degree* level maths found them clear to interpret, whereas the responses of the other two groups were mostly split between ‘eventually understood’ and ‘clear’.

### 3.3 Questions 3 and 4: are the following statements clear?

**Fig. 8:**
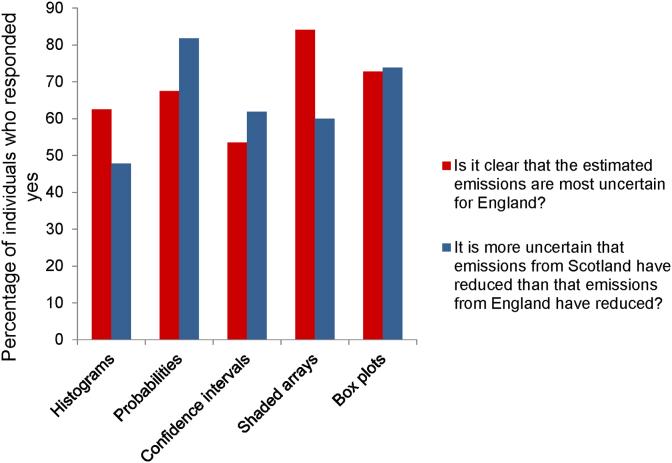
Bar charts showing how participants responded to Questions 3 and 4.

Fig. 8 summarises responses to Questions 3 and 4. For both, there were significant differences in how participants responded to the methods when groups were pooled (Tables S6–S9). Shaded arrays proved the best method for interpreting the statement in Question 3 and confidence intervals the worst. There was a significant difference in how the professional groups (but not mathematical groups) responded on Boxplots for both questions: more research scientists than expected thought the message was clear. For Question 4, more than 70% of respondents were able to interpret the uncertainties in the reductions in emissions using probabilities or boxplots, other methods proved less successful.

### 3.4 Question 5: for each scenario, which method or combination of methods do you think should be used to communicate uncertainty to your professional group?

Most participants (72%) wanted a combination of methods used to communicate the uncertainty in estimated emissions. The government and policy group favoured combinations of words, boxplots and shaded arrays. Similarly the industry group favoured combinations of words, boxplots, shaded arrays and confidence intervals. Research scientists selected confidence intervals and boxplots. The results on the environment group were inconclusive.

### 3.5 Question 6: which method or combination of methods would you use to communicate uncertainty?

Our participants told us that they were required to communicate the uncertainty in estimated greenhouse gas emissions to farmers, policy makers and research scientists. Most (62%) thought that more than one method should be used to do this. For policy makers participants preferred to use combinations with shaded arrays or boxplots, for communicating uncertainty to farmers participants generally opted for words or shaded arrays, and for communicating uncertainty to research scientists participants favoured confidence intervals and boxplots.

### 3.6 Additional questions

Out of those who responded 56% did not think that colour aided the interpretation of the IPCC phrases for communicating uncertainty. There were no strong differences in preferences between professional groups.

**Fig. 9:**
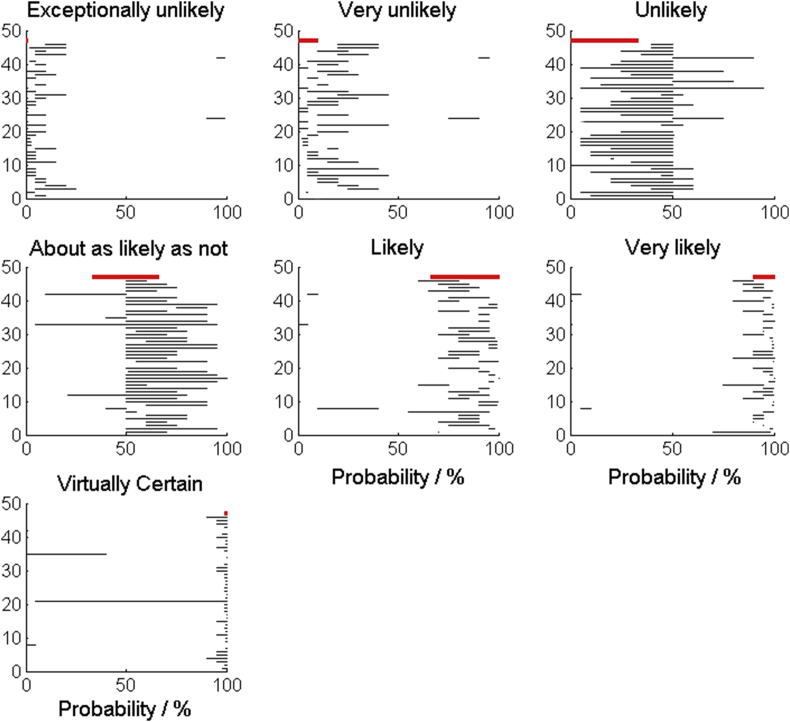
The black lines show the ranges of values that participants mapped the calibrated phrases to. The red line shows the range that the IPCC define for each phrase.

Broadly, our participants interpreted the IPCC phrases for communicating the probability of an outcome reasonably, with one or two exceptions (Fig. 9). The least successful phrase was ‘about as likely as not’. This phrase maps to a probability interval of 33–66%, whereas most of our participants mapped it to intervals that did not extend below 50% (i.e. they associated the phrase with a more likely outcome).

In response to our question on whether it was helpful to have confidence intervals expressed in the same units as the mean or as a percentage of the mean we found research scientists were split between only wanting confidence intervals expressed in units of the mean (14 individuals) and wanting both (19 individuals) (8 did not respond). Almost all participants from the other three professional groups wanted both methods used.

Of the methods tested, 47% of participants said that they liked boxplots the best, 17% liked shaded arrays the best, 11% liked confidence intervals the best, 10% words, 9% histograms and 6% probabilities.

### 3.7 Additional comments

The additional comments given were mixed, but agreed with analysis of the closed form questions. The verbal scale was criticised for being too vague and open to misinterpretation, however, some felt that they were useful and did serve a purpose. There was a call for the mapping of calibrated phrases to probabilities to be explicitly stated in the text, agreeing with the work done by Budescu et al. (2009). The numerical methods (probabilities and confidence intervals) were criticised by some for being difficult to interpret whereas others found these more quantitative approaches clear. This difference of opinion was driven by differences in mathematical background. Shaded arrays received the fewest negative comments. Several respondents commented that they were easy to interpret and represented the concept of uncertainty well, stating that they gave a ‘good starting point’ for interpretation. Others struggled to understand the significance of the shading. Histograms received the most negative comments and were criticised for being confusing. In particular the respondents found it difficult to interpret comparisons between two or more. Some commented that boxplots were clear and familiar and by far the best approach, whereas others were concerned that not everyone would understand what the various parts of the box represented (i.e. the quartiles and median marks). This difference of opinion was driven by mathematical background. Across the methods there was a call for all of the methods used to be better annotated, which essentially meant that methods should be combined.

## 4 Discussion

The six methods for communicating uncertainty that we tested convey a range of detail, and their interpretation relies on varying levels of numeracy. At one extreme, the verbal scale requires little numeracy but also conveys little detail and is subject to inconsistent interpretations between individuals. We used the verbal scale published by the IPCC in our study. Budescu et al. (2009) reported substantial differences in the way people interpreted the scale and the intended meaning. Harris and Corner (2011) showed that the severity of the outcome can also affect the way that the verbal scale can be interpreted, with expressions that refer to a severe outcome being interpreted as denoting higher probabilities than those that refer to more neutral outcomes. In our study the verbal scale was criticised for communicating too little detail and being open to misinterpretation. Several of our participants commented that it did have value, however, and suggested that it would be helpful to present the associated numerical probabilities alongside the calibrated phrases. This accords with the recommendations of Budescu et al. (2009) and Harris and Corner (2011), who found that including the numerical probability range in the text along with the calibrated phrase improved interpretation. Our results on how our participants mapped the calibrated phrases to a scale of probabilities showed that in most cases the mapping was broadly correct. Similar to Budescu et al. (2009), we found that inconsistencies between the perceived probability ranges and the intended probability range became larger for the more extreme terms. The least successful phrase was ‘about as likely as not’, which confused our participants who generally interpreted it as meaning “more likely than not” (i.e. a probability somewhere over 50%). We found that this phrase was the one which most irritated our participants because of its vagueness.

Numerical methods enable one to assign some level of precision to uncertainty, but the ability to interpret the message depends on the numeracy of the end-user. Similarly, we found differences in how participants with different levels of familiarity with statistical methods wanted confidence intervals presented. Participants who did not work in research wanted to see confidence intervals expressed as both units of the estimated value and as a percentage of the expected value. Many research scientists (who are likely to be more familiar with this method) thought this unnecessary, opting for only expressing confidence intervals in the units of the estimated value. Representing uncertainty as a percentage of the mean could have a biasing effect on the interpretation of results when comparing the uncertainty in emissions from several sources: a 10% uncertainty on 60 kt N_2_O is quite different to a 10% uncertainty on 6 kt N_2_O. This is similar to the concept of denominator neglect (Spiegelhalter et al., 2011). Therefore, if one is to present results as a percentage of the expected value it would be wise to also present the uncertainty in units of the estimated value.

Similar to confidence intervals and probability intervals, histograms and boxplots give more detailed information using statistical concepts. This means that their interpretation does, to some extent, depend on the familiarity of the user with these methods. In our study, boxplots were reported to convey an appropriate level of information and to do this clearly, but a more detailed examination showed that this result was driven by the responses from research scientists who were largely more familiar with this method of displaying information and most of whom (>85%) were educated to at least the *higher* level maths. Mathematical background also had a significant effect on the responses to confidence intervals with the more numerate respondents viewing them more favourably. Boxplots, confidence intervals and histograms are typically used to display data, whereas in our study they display the Monte Carlo simulation output which represents the uncertainty in a single datum. The link between the Monte Carlo output and the uncertainty in a single value may be hard for many to make. Histograms proved unpopular across the groups and we believe that this is because they are less intuitive than the other methods we tested. We found that some participants had misinterpreted the relative height of two histograms as conveying the relative size of emissions while it actually conveys differences in variability. This accords with the findings of Morgan and Henrion (1990) who found that people often find it difficult to interpret PDFs. Our third graphical method, shaded arrays, proved to be more intuitive and relied less on numerical ability or familiarity with statistical concepts. We found that shaded arrays proved the best method for portraying the uncertainty in emissions (see results from Question 3, Fig. 8) and we believe that this is because their simple visual interpretation of uncertainty is relatively easy to understand, regardless of mathematical background.

There were few significant differences in how participants responded according to scenario. The most notable being that a significantly larger number of participants did not think confidence intervals represented the information needed to compare emissions to a given threshold value, and similarly a significantly larger number of participants did not think probabilities represented the emission rates given in Scenario A satisfactorily.

We found that our more numerate research scientists were generally content to have uncertainty simply portrayed with boxplots and confidence intervals. Other groups were keen to have a combination of methods used, favouring a mixture of the more intuitive words and shaded arrays used in combination with boxplots. Presenting uncertainty with these sorts of combinations allows the user to progress to the more quantitative description should they so wish to, and can give confidence that interpretations are correct. This is known as progressive disclosure (Kloprogge et al., 2007). Our results from question 6, where we asked which methods the participants would use to communicate to ‘other groups’ suggested a similar pattern. Participants favoured using a mixture of words, shaded arrays and boxplots for groups that are not regularly exposed to statistics, and confidence intervals or boxplots for communicating with research scientists.

## 5 Conclusion and recommendation

**Fig. 10:**
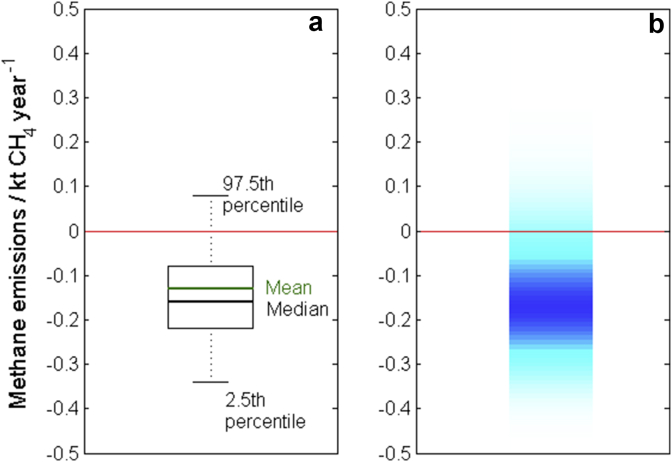
The trend in emissions of CH_4_ from agriculture in Wales between 1990 and 2010 shown using (a) a boxplot with the expected value (mean), median, 2.5th and 97.5th percentiles annotated on the graph and (b) a shaded array where the intensity of colour indicates the frequency of each observation with darker shading indicating a larger probability of observing that value. The expected value is −0.13 with 95% confidence interval given by [−0.34, 0.08]. The red lines mark the zero line. (For interpretation of the references to colour in this figure legend, the reader is referred to the web version of this article.).

The methods chosen to communicate uncertainty in estimates of greenhouse gas emissions should be influenced by professional and mathematical background of the target audience. In our study we found that research scientists tended to be familiar with boxplots and confidence intervals and so found these methods straightforward to interpret. We propose that boxplots annotated with summary statistics such as mean, median, 2.5th and 97.5th percentiles provide a sound method for communicating uncertainty to these individuals (see Fig. 10a). End-users from other groups may not be so familiar with these methods and so a combination of intuitive methods such as calibrated phrases and shaded arrays with numerical methods would be better suited. Ideally uncertainty should be presented to these individuals in such a way that they can form an initial impression from verbal and visual information, and then progress to the more quantitative description should they so wish. For example, the use of key phrases in text alongside a shaded array either annotated with some summary statistics quoted in the figure caption (for example Fig. 10b), or with an annotated boxplot presented in the appendices.

## Figures and Tables

**Table 1:** The verbal likelihood scale developed by the IPCC (2010) with the colour coding developed by Kloprogge et al. (2007).

Calibrated phrase	Likelihood of outcome	Colour coding
Virtually certain	99–100% probability	Green
Very likely	90–100% probability	Green
Likely	66–100% probability	Green
About as likely as not	33–66% probability	Amber
Unlikely	0–33% probability	Red
Very unlikely	0–10% probability	Red
Exceptionally unlikely	0–1% probability	Red

**Table 2a:** The contingency table showing how many individuals selected a given response to Question 1. The table is presented according to scenario (A—D) and method of communication.

	Verbal scale				Probabilities				Confidence intervals				Histograms				Shaded arrays				Boxplots			
	A	B	C	D	A	B	C	D	A	B	C	D	A	B	C	D	A	B	C	D	A	B	C	D
Not enough	51	45	44	40	26	18	24	17	7	17	7	8	19	13	18	13	33	22	22	27	7	7	9	12
Enough	11	17	18	22	35	25	30	35	33	35	47	41	33	41	33	37	28	40	39	30	44	50	46	39
Too much	1	1	1	1	1	19	8	9	21	9	7	12	9	7	10	10	1	0	0	1	12	6	8	10

**Table 2b:** The contingency table showing how many individuals selected a given response to Question 1. The table is presented according to scenario (A—D) and is pooled by method of communication.

	Scenario			
	A	B	C	D
Not enough	143	122	124	117
Enough	184	208	213	204
Too much	45	42	34	43

**Table 2c:** The Scenario A sub-table showing how many individuals selected a given response to Question 1.

	Verbal scale	Probabilities	Confidence intervals	Histograms	Shaded arrays	Boxplots
Not enough	51	26	7	19	33	7
Enough	11	35	33	33	28	44
Too much	1	1	21	9	1	12

**Table 3a:** The contingency table showing how many individuals selected a given response to Question 1 on the verbal scale. The table is presented according to scenario (A—D) and mathematical group (lower, higher, degree level).

	Lower				Higher				Degree			
	A	B	C	D	A	B	C	D	A	B	C	D
Not enough	12	11	13	10	17	13	10	13	22	21	21	17
Enough	7	8	6	9	1	5	8	5	3	4	4	8
Too much	0	0	0	0	0	0	0	0	1	1	1	1

**Table 3b:** The contingency table showing how many individuals selected a given response to Question 1 on the verbal scale. The table is presented according to scenario (A—D) and is pooled by mathematical group.

	Scenario			
	A	B	C	D
Not enough	51	45	44	40
Enough	11	17	18	22
Too much	1	1	1	1

**Table 3c:** The Scenario A sub-table showing how many individuals selected a given response to Question 1.

	Lower	Higher	Degree
Not enough	12	17	22
Enough	7	1	3
Too much	0	0	1

**Table 4:** Analysis of question 1 according to method and scenario, p-values <0.05 are highlighted by a single star, those <0.01 with two stars, and those <0.001 with three.

	Pearson χ^2^-value	p-value
Full table	363.57	<0.001***
Table pooled by methods	7.04	0.315
Scenario A sub-table	122.71	<0.001***
Scenario B sub-table	95.22	<0.001***
Scenario C sub-table	74.14	<0.001***
Scenario D sub-table	59.32	<0.001***

**Table 5:** Analysis of question 2 according to method and scenario, p-values <0.05 are highlighted by a single star, those <0.01 with two stars, and those <0.001 with three.

	Pearson χ^2^-value	p-value
Full table	258.55	<0.001***
Table pooled by methods	8.41	0.772
Scenario A sub-table	77.79	<0.001***
Scenario B sub-table	58.24	<0.001***
Scenario C sub-table	61.77	<0.001***
Scenario D sub-table	51.76	<0.001***

## Box 2 The material used to test probabilities as a method for communicating uncertainty.

Scenario A : *Comparing emissions of nitrous oxide from different countries*

There is a 95% probability that emissions of N_2_O from agriculture in 2010 lay between:

**Table:**

Country	Emissions of nitrous oxide/kt N_2_O year^−1^
England	20 and 120
Wales	3 and 17
Scotland	5 and 26
Northern Ireland	3 and 15

Scenario B : *Comparing emissions of methane to a given threshold value*

In our example, the arbitrarily chosen threshold value for methane emissions is 130 kt CH_4_ year^−1^.

• There is a less than 0.01% probability that emissions of CH_4_ from English agriculture are less than the threshold, and so a greater than 99.99% probability that they are larger.
• There is an 87% probability that emissions of CH_4_ from Welsh agriculture are less than the threshold, and so a 13% probability that they are larger.
• There is a 21% probability that emissions of CH_4_ from Scottish agriculture are less than the threshold, and so a 79% probability that they are larger.
• There is a 94% probability that emissions of CH_4_ from Northern Irish agriculture are less than the threshold, and so a 6% probability that they are larger.

Scenario C : *Assessing changes in methane emissions over time*

The trend in emissions from 1990 to 2010 is expressed as the percentage change in emissions from the 1990 base year. The table below gives the percentage probabilities that emissions of CH_4_ from agriculture have diminished or increased.

**Table:**

Country	Probability emissions diminished/%	Probability emissions increased/%
England	98.9	0.11
Wales	91.6	8.4
Scotland	94.7	5.3
Northern Ireland	47.3	52.7

Scenario D : *Assessing mitigation methods for the best opportunity to reduce emissions of nitrous oxide*

Nitrous oxide emissions from English grasslands in 2010 were assessed under conventional management and under management with mitigation. There is a 99.7% probability that the mitigation strategy results in fewer emissions and so a 0.3% probability that it does not.

## Box 3 The material used to test confidence intervals as a method for communicating uncertainty.

Scenario A : *Comparing emissions of nitrous oxide from different countries*

Estimated emissions of N_2_O from agriculture in 2010, with the 95% confidence interval, are listed below. The 95% confidence interval is expressed in both kt N_2_O year^−1^ and as a percentage of the mean.

**Table:**

Country	Mean/kt N_2_O year^−1^	95% confidence interval	
		/kt N_2_O year^−1^	Percentage of mean
England	60	(20, 120)	(−60%, +100%)
Wales	9	(3, 17)	(−60%, +88%)
Scotland	13	(5, 26)	(−60%, +100%)
Northern Ireland	8	(3, 15)	(−60%, +88%)

Scenario B : *Comparing emissions of methane to a given threshold value*

Estimated emissions of CH_4_ from agriculture in 2010, with the 95% confidence interval compared to the arbitrarily chosen threshold value of 130 kt CH_4_ year^−1^.

**Table:**

Country	Mean/kt CH_4_ year^−1^	95% confidence interval/kt CH_4_ year^−1^
England	474	(415, 544)
Wales	118	(99, 141)
Scotland	140	(118, 166)
Northern Ireland	116	(100, 134)

Scenario C : *Assessing changes in methane emissions over time*

The trend in emissions from 1990 to 2010 with their 95% confidence intervals. The trend in emissions is expressed as the percentage change in emissions from the 1990 base year.

**Table:**

Country	Trend/%	95% confidence interval/%
England	−26	(−38, −11)
Wales	−15	(−34, −8)
Scotland	−16	(−33, −4)
Northern Ireland	1	(−17, 21)

Scenario D : *Assessing mitigation methods for the best opportunity to reduce emissions of nitrous oxide*

Nitrous oxide emissions from English grasslands in 2010 were assessed under conventional management and under management with mitigation. The expected reduction in emissions due to mitigation was 30 kt N_2_O year^−1^ with 95% confidence interval (13, 47) kt N_2_O year^−1^.
