# Supplementary material for: Communicating the uncertainty in estimated greenhouse gas emissions from agriculture
Source: J Environ Manage. 2015 Sep 1;160:139–53. doi: 10.1016/j.jenvman.2015.05.034 (PMC4521119; doi:10.1016/j.jenvman.2015.05.034)
Supplement: Supplementary file 1 [file mmc1.docx]

Table S1: The four central questions posed in the questionnaire. Questions 3 and 4 were not asked in the context of the verbal scale as there insufficient information to given an answer (see Box 1).

**Q1: Is the information presented on uncertainty sufficient for your needs?**

|  | Scenario | | | |
| --- | --- | --- | --- | --- |
|  | A: different sources | B: reference value | C: change over time | D: mitigation |
| Not enough information |  |  |  |  |
| Shows the information I want |  |  |  |  |
| More information than I want or need |  |  |  |  |

**Q2: Is this method of representing uncertainty straightforward to interpret?**

|  | Scenario | | | |
| --- | --- | --- | --- | --- |
|  | A: different sources | B: reference value | C: change over time | D: mitigation |
| I find it impossible to understand |  |  |  |  |
| I understand most of what has been presented but it took me a while to get it |  |  |  |  |
| I think this method could be misinterpreted (please expand below) |  |  |  |  |
| Good but needs more explanation (please expand below) |  |  |  |  |
| The message is clear |  |  |  |  |

**Q3: Is the following statement about Scenario A clear from the poster?**

|  | Yes | No |
| --- | --- | --- |
| The estimated emissions are most uncertain for England |  |  |

**Q4: Is the following statement about Scenario C clear from the poster?**

|  | Yes | No |
| --- | --- | --- |
| It is more uncertain that emissions from Scotland have reduced than that emissions from England have reduced. |  |  |

Table S2: Analysis of question 1 according to professional group and scenario, p-values <0.05 are highlighted by a single star, those <0.01 with two stars, and those < 0.001 with three.

|  | Pearson χ^2^-value | p-value |
| --- | --- | --- |
| *Verbal scale* |  |  |
| Full Table | 28.91 | 0.497 |
| Table pooled by groups | 5.02 | 0.549 |
| Scenario A sub-table | 2.48 | 0.788 |
| Scenario B sub-table | 9.50 | 0.199 |
| Scenario C sub-table | 4.33 | 0.541 |
| Scenario D sub-table | 6.93 | 0.317 |
|  |  |  |
| *Histograms* |  |  |
| Full Table | 32.11 | 0.347 |
| Table pooled by groups | 3.82 | 0.697 |
| Scenario A sub-table | 10.35 | 0.089 |
| Scenario B sub-table | 5.47 | 0.474 |
| Scenario C sub-table | 4.12 | 0.685 |
| Scenario D sub-table | 8.67 | 0.191 |
|  |  |  |
| *Probabilities* |  |  |
| Full Table | 38.20 | 0.127 |
| Table pooled by groups | 22.69 | 0.002** |
| Scenario A sub-table | 1.21 | 0.925 |
| Scenario B sub-table | 3.42 | 0.811 |
| Scenario C sub-table | 7.55 | 0.305 |
| Scenario D sub-table | 4.37 | 0.645 |
|  |  |  |
| *Confidence intervals* |  |  |
| Full Table | 53.97 | 0.005** |
| Table pooled by groups | 19.70 | <0.001*** |
| Scenario A sub-table | 4.60 | 0.648 |
| Scenario B sub-table | 10.55 | 0.07 |
| Scenario C sub-table | 18.40 | 0.005** |
| Scenario D sub-table | 5.21 | 0.538 |
|  |  |  |
| *Shaded arrays* |  |  |
| Full Table | 19.30 | 0.789 |
| Table pooled by groups | 8.19 | 0.222 |
| Scenario A sub-table | 3.94 | 0.608 |
| Scenario B sub-table | 2.09 | 0.583 |
| Scenario C sub-table | 0.44 | 0.954 |
| Scenario D sub-table | 3.66 | 0.582 |
|  |  |  |
| *Box plots* |  |  |
| Full Table | 35.83 | 0.184 |
| Table pooled by groups | 5.51 | 0.446 |
| Scenario A sub-table | 3.68 | 0.759 |
| Scenario B sub-table | 13.51 | 0.044* |
| Scenario C sub-table | 8.19 | 0.202 |
| Scenario D sub-table | 6.76 | 0.341 |

Table S3: Analysis of question 1 according to mathematical background group and scenario (note that the table pooled by groups in each case is the same as in Table 5), p-values <0.05 are highlighted by a single star, those <0.01 with two stars, and those < 0.001 with three.

|  | Pearson χ^2^-value | p-value |
| --- | --- | --- |
| *Verbal scale* |  |  |
| Full Table | 26.20 | 0.224 |
| Table pooled by groups | 5.02 | 0.549 |
| Scenario A sub-table | 8.69 | 0.024* |
| Scenario B sub-table | 5.14 | 0.222 |
| Scenario C sub-table | 5.63 | 0.177 |
| Scenario D sub-table | 3.24 | 0.594 |
|  |  |  |
| *Histograms* |  |  |
| Full Table | 14.63 | 0.886 |
| Table pooled by groups | 3.82 | 0.697 |
| Scenario A sub-table | 4.46 | 0.360 |
| Scenario B sub-table | 3.72 | 0.478 |
| Scenario C sub-table | 2.57 | 0.687 |
| Scenario D sub-table | 0.57 | 0.976 |
|  |  |  |
| *Probabilities* |  |  |
| Full Table | 34.56 | 0.045* |
| Table pooled by groups | 22.69 | 0.002** |
| Scenario A sub-table | 2.95 | 0.669 |
| Scenario B sub-table | 4.16 | 0.413 |
| Scenario C sub-table | 0.66 | 0.949 |
| Scenario D sub-table | 3.84 | 0.445 |
|  |  |  |
| *Confidence intervals* |  |  |
| Full Table | 27.90 | 0.184 |
| Table pooled by groups | 19.70 | < 0.001*** |
| Scenario A sub-table | 1.24 | 0.899 |
| Scenario B sub-table | 0.88 | 0.922 |
| Scenario C sub-table | 3.63 | 0.514 |
| Scenario D sub-table | 4.08 | 0.421 |
|  |  |  |
| *Shaded arrays* |  |  |
| Full Table | 45.19 | 0.002** |
| Table pooled by groups | 8.19 | 0.222 |
| Scenario A sub-table | 13.61 | 0.004** |
| Scenario B sub-table | 3.11 | 0.368 |
| Scenario C sub-table | 4.60 | 0.142 |
| Scenario D sub-table | 12.08 | 0.008** |
|  |  |  |
| *Box plots* |  |  |
| Full Table | 22.98 | 0.368 |
| Table pooled by groups | 5.51 | 0.446 |
| Scenario A sub-table | 7.96 | 0.073 |
| Scenario B sub-table | 5.11 | 0.301 |
| Scenario C sub-table | 2.14 | 0.758 |
| Scenario D sub-table | 1.47 | 0.862 |

Table S4: Analysis of question 2 according to professional group and scenario, p-values <0.05 are highlighted by a single star, those <0.01 with two stars, and those < 0.001 with three.

|  | Pearson χ^2^-value | p-value |
| --- | --- | --- |
| *Verbal scale* |  |  |
| Full Table | 68.04 | 0.224 |
| Table pooled by groups | 5.34 | 0.952 |
| Scenario A sub-table | 14.08 | 0.275 |
| Scenario B sub-table | 17.63 | 0.114 |
| Scenario C sub-table | 11.73 | 0.463 |
| Scenario D sub-table | 17.99 | 0.103 |
|  |  |  |
| *Histograms* |  |  |
| Full Table | 87.86 | 0.012* |
| Table pooled by groups | 8.90 | 0.727 |
| Scenario A sub-table | 16.99 | 0.129 |
| Scenario B sub-table | 20.40 | 0.049* |
| Scenario C sub-table | 19.44 | 0.068 |
| Scenario D sub-table | 20.46 | 0.061 |
|  |  |  |
| *Probabilities* |  |  |
| Full Table | 60.84 | 0.449 |
| Table pooled by groups | 8.46 | 0.756 |
| Scenario A sub-table | 12.34 | 0.408 |
| Scenario B sub-table | 16.79 | 0.169 |
| Scenario C sub-table | 11.72 | 0.473 |
| Scenario D sub-table | 11.46 | 0.506 |
|  |  |  |
| *Confidence intervals* |  |  |
| Full Table | 72.71 | 0.119 |
| Table pooled by groups | 11.17 | 0.553 |
| Scenario A sub-table | 14.70 | 0.252 |
| Scenario B sub-table | 9.42 | 0.710 |
| Scenario C sub-table | 22.36 | 0.033* |
| Scenario D sub-table | 18.64 | 0.094 |
|  |  |  |
| *Shaded arrays* |  |  |
| Full Table | 53.14 | 0.713 |
| Table pooled by groups | 3.29 | 0.991 |
| Scenario A sub-table | 9.85 | 0.674 |
| Scenario B sub-table | 16.37 | 0.169 |
| Scenario C sub-table | 11.86 | 0.472 |
| Scenario D sub-table | 12.18 | 0.438 |
|  |  |  |
| *Box plots* |  |  |
| Full Table | 105.06 | 0.003** |
| Table pooled by groups | 8.73 | 0.741 |
| Scenario A sub-table | 23.86 | 0.032*** |
| Scenario B sub-table | 28.26 | 0.005** |
| Scenario C sub-table | 22.27 | 0.050* |
| Scenario D sub-table | 18.77 | 0.082 |

Table S5: Analysis of question 2 according to mathematical background group and scenario (note that the table pooled by groups in each case is the same as in Table 8), p-values <0.05 are highlighted by a single star, those <0.01 with two stars, and those < 0.001 with three.

|  | Pearson χ^2^-value | p-value |
| --- | --- | --- |
| *Verbal scale* |  |  |
| Full Table | 33.78 | 0.893 |
| Table pooled by groups | 5.34 | 0.952 |
| Scenario A sub-table | 8.03 | 0.454 |
| Scenario B sub-table | 8.28 | 0.406 |
| Scenario C sub-table | 5.67 | 0.697 |
| Scenario D sub-table | 5.38 | 0.724 |
|  |  |  |
| *Histograms* |  |  |
| Full Table | 58.55 | 0.064 |
| Table pooled by groups | 8.90 | 0.727 |
| Scenario A sub-table | 10.76 | 0.233 |
| Scenario B sub-table | 12.59 | 0.136 |
| Scenario C sub-table | 11.55 | 0.177 |
| Scenario D sub-table | 14.03 | 0.079 |
|  |  |  |
| *Probabilities* |  |  |
| Full Table | 35.35 | 0.854 |
| Table pooled by groups | 8.46 | 0.756 |
| Scenario A sub-table | 5.90 | 0.712 |
| Scenario B sub-table | 5.59 | 0.734 |
| Scenario C sub-table | 6.49 | 0.638 |
| Scenario D sub-table | 9.62 | 0.294 |
|  |  |  |
| *Confidence intervals* |  |  |
| Full Table | 59.94 | 0.037* |
| Table pooled by groups | 10.32 | 0.63 |
| Scenario A sub-table | 15.06 | 0.059 |
| Scenario B sub-table | 12.52 | 0.123 |
| Scenario C sub-table | 18.32 | 0.016* |
| Scenario D sub-table | 4.58 | 0.847 |
|  |  |  |
| *Shaded arrays* |  |  |
| Full Table | 34.55 | 0.873 |
| Table pooled by groups | 3.29 | 0.991 |
| Scenario A sub-table | 8.70 | 0.387 |
| Scenario B sub-table | 6.39 | 0.616 |
| Scenario C sub-table | 10.38 | 0.213 |
| Scenario D sub-table | 5.26 | 0.777 |
|  |  |  |
| *Box plots* |  |  |
| Full Table | 75.60 | 0.001*** |
| Table pooled by groups | 8.73 | 0.741 |
| Scenario A sub-table | 18.99 | 0.005** |
| Scenario B sub-table | 20.97 | 0.006** |
| Scenario C sub-table | 21.21 | 0.002** |
| Scenario D sub-table | 8.41 | 0.394 |

Table S6: Analysis of Question 3 according to method and professional group, p-values <0.05 are highlighted by a single star, those <0.01 with two stars, and those < 0.001 with three.

|  | Pearson χ^2^-value | p-value |
| --- | --- | --- |
| Full Table | 28.32 | 0.073 |
| Table pooled by groups | 12.09 | 0.017* |
| Histogram sub-table | 2.60 | 0.601 |
| Probabilities sub-table | 2.76 | 0.458 |
| Confidence intervals sub-table | 1.52 | 0.730 |
| Shaded arrays sub-table | 2.94 | 0.419 |
| Boxplots sub-table | 8.85 | 0.030* |

Table S7: Analysis of Question 3 according to method and mathematical group (note that the table pooled by groups in each case is the same as in Table 10), p-values <0.05 are highlighted by a single star, those <0.01 with two stars, and those < 0.001 with three.

|  | Pearson χ^2^-value | p-value |
| --- | --- | --- |
| Full Table | 26.72 | 0.022* |
| Table pooled by groups | 12.09 | 0.017* |
| Histogram sub-table | 4.94 | 0.100 |
| Probabilities sub-table | 5.86 | 0.056 |
| Confidence intervals sub-table | 2.13 | 0.411 |
| Shaded arrays sub-table | 0.64 | 0.874 |
| Boxplots sub-table | 0.36 | 0.875 |

Table S8: Analysis of Question 4 according to method and professional group, p-values <0.05 are highlighted by a single star, those <0.01 with two stars, and those < 0.001 with three.

|  | Pearson χ^2^-value | p-value |
| --- | --- | --- |
| Full Table | 31.71 | 0.027* |
| Table pooled by groups | 16.12 | 0.002** |
| Histogram sub-table | 3.13 | 0.422 |
| Probabilities sub-table | 3.32 | 0.305 |
| Confidence intervals sub-table | 2.85 | 0.449 |
| Shaded arrays sub-table | 3.65 | 0.338 |
| Boxplots sub-table | 8.62 | 0.028* |

Table S9: Analysis of Question 4 according to method and professional group (note that the table pooled by groups in each case is the same as in Table 12) , p-values <0.05 are highlighted by a single star, those <0.01 with two stars, and those < 0.001 with three.

|  | Pearson χ^2^-value | p-value |
| --- | --- | --- |
| Full Table | 20.43 | 0.120 |
| Table pooled by groups | 16.12 | 0.002** |
| Histogram sub-table | 0.00 | 1.000 |
| Probabilities sub-table | 0.85 | 0.784 |
| Confidence intervals sub-table | 1.54 | 0.526 |
| Shaded arrays sub-table | 0.25 | 0.900 |
| Boxplots sub-table | 2.48 | 0.304 |
